# High serum vascular endothelial growth factor C predicts better relapse-free survival in early clinically node-negative breast cancer

**DOI:** 10.18632/oncotarget.25577

**Published:** 2018-06-15

**Authors:** José Maañón, Diego Perez, Alejandro Rhode, Gonzalo Callejón, Francisco Rivas-Ruiz, Elisabeth Perez-Ruiz, Isabel Rodrigo, Belén Ramos, Francisco Medina, Rosa Villatoro, Maximino Redondo, Antonio Rueda

**Affiliations:** ^1^ Obstetrics and Gynecology Unit, Breast Cancer Unit, Hospital Costa de Sol, Málaga University, Málaga, Spain; ^2^ Medical Oncology Unit, Breast Cancer Unit, Hospital Costa de Sol, (REDISSEC), Marbella, Málaga, Spain; ^3^ Obstetrics and Gynecology Unit, Breast Cancer Unit, Hospital Costa de Sol, Marbella, Málaga, Spain; ^4^ Clinical Analysis Laboratory Unit, Hospital Costa de Sol, Marbella, Málaga, Spain; ^5^ Support for Research Unit, Hospital Costa de Sol, (REDISSEC), Marbella, Málaga, Spain; ^6^ Pathology Unit, Breast Cancer Unit, Hospital Costa de Sol, Marbella, Málaga, Spain; ^7^ Radiology Unit, Breast Cancer Unit, Hospital Costa de Sol, Marbella, Málaga, Spain; ^8^ General and Digestive Surgery Unit, Breast Cancer Unit, Hospital Costa de Sol, (REDISSEC), Marbella, Málaga, Spain; ^9^ Hospital Tumor Registry, Hospital Costa de Sol, (REDISSEC), Marbella, Málaga, Spain

**Keywords:** breast cancer, sentinel lymph node biopsy, clinically node negative, VEGF-C, relapse-free survival

## Abstract

A recent meta-analysis indicated that higher tumoral expression of vascular endothelial growth factor C (VEGF-C) was related to poorer relapse-free and overall survival in breast cancer patients. However, a retrospective study found that higher circulating VEGF-C levels were associated with better survival in breast cancer patients. In 2009, we initiated a prospective study to determine the utility of preoperative serum VEGF-C levels for predicting the risk of sentinel lymph node involvement in early breast cancer and to assess serum VEGF-C levels as a prognostic factor for relapse-free and overall survival. We analyzed serum samples from 174 patients with early breast cancer who underwent sentinel lymph node biopsies. VEGF-C levels were determined using an ELISA. Serum VEGF-C levels were normally distributed, with a median value of 6561.5 pg/mL, and did not correlate with any other clinical or pathological variables. During a median follow-up period of 58 months, the five-year relapse-free survival rate was higher in patients with VEGF-C levels above the median than in patients with lower levels (95.3% vs. 85.9%, *p* < 0.04). No association was found between VEGF-C levels and overall survival. Our study demonstrates that the prognosis was better for early breast cancer patients with high serum VEGF-C levels.

## INTRODUCTION

The invasion of solid tumors into the lymph vessels and the consequent generation of lymph node metastases require the growth of new lymphatic vessels (lymphangiogenesis). The mechanisms by which tumors promote lymphangiogenesis remain unclear, but the development of molecular biology has led to the discovery of growth factors that promote lymphangiogenesis and solid tumor dissemination by lymphatic pathways. Specifically, vascular endothelial growth factors C and D (VEGF-C and VEGF-D) and the membrane receptor vascular endothelial growth factor receptor-3 (VEGFR-3) are strongly associated with these processes and have been studied extensively [1]. The expression of VEGF-C (and to a lesser extent that of VEGF-D) has been shown to correlate strongly with the risk of lymph node metastasis in over 30 studies of different malignancies, including lung, breast and colon cancers [2–4].

In breast cancer, greater expression of VEGF-C (measured by immunohistochemistry in tumor biopsies) is related to poorer relapse-free survival (RFS) and overall survival (OS). A recent meta-analysis that included 21 studies and 2828 patients with breast cancer reported hazard ratios (HRs) of 1.87 (95% confidence interval [CI] 1.25–2.79, *p* = 0.001) for RFS and 1.96 (95% CI 1.15–3.31, *p* = 0.001) for OS in patients with high tumor VEGF-C expression [5]. However, the cut-off values used to define high tumor VEGF-C expression varied considerably among the studies.

The measurement of serum VEGF-C levels is minimally invasive and can be performed at different times during the course of breast cancer. If serum VEGF-C levels were found to correlate with the risk of axillary nodal spread, the measurement of serum VEGF-C levels would offer a simple, risk-free means of predicting lymph node status. This would enable the optimal use of resources such as operating room times, and would reduce the time interval between the histological diagnosis and first surgical treatment for breast cancer. In September 2009, our group initiated a prospective study to determine the value of preoperative serum VEGF-C levels and predict the risk of axillary lymph node involvement in female early breast cancer patients undergoing local surgical treatment and sentinel lymph node biopsy (SLNB). Our published results revealed that serum VEGF-C levels could not be used to predict sentinel lymph node status [6]. In this study, a secondary objective was to assess serum VEGF-C levels as a prognostic factor for RFS and OS.

To date, two published retrospective studies have described the association of circulating VEGF-C levels with survival in breast cancer patients. Both studies included stage I–III newly diagnosed patients. In the first study, no correlation was found between plasma VEGF-C levels and OS in 122 patients [7]. However, in the other study, Gisterek *et al.* reported that serum VEGF-C levels above 1784.699 pg/mL predicted better four-year OS than lower levels in 349 patients (93% *vs.* 82%, *p* < 0.007) [8]. Here, we provide the results of the first prospective study correlating RFS with preoperative serum VEGF-C levels in early breast cancer patients.

## RESULTS

In total, 181 patients were included during the study period (September 2009 to November 2012. Seven patients (4%) did not undergo SLNB due to problems associated with the technique (non-migration of the tracer or failure to achieve intraoperative localization) and underwent ALND concurrently with the primary surgery. Thus, only the 174 patients undergoing SLNB were included in the analysis. The median age of the study patients was 57 years (32–79 years). Over half of the patients were postmenopausal at diagnosis, and the most common histological type was ductal carcinoma. Most of the patients had tumors with good prognoses: over half had grade I-II tumors smaller than 2 cm with no vascular invasion and were positive for hormone receptors. A KI67 index exceeding 14% was observed in 62.1% of the participating patients (see Table 1).

**Table 1:** Patient characteristics

Patient characteristics		
	Median	
Age	57 years	32–79
	*N* (174)	(%)
Menopausal status		
Post	115	66
Pre	59	34
Histological subtype		
Ductal	137	78.7
Lobular	12	6.9
Other	25	14.4
T size (TNM)		
Tmic	4	0.7
T1a	10	3.5
T1b	35	19.9
T1c	95	58.2
T2	30	17.7
Histological grade		
Grade I	48	27.5
Grade II	66	37.9
Grade III	58	33.3
Unknown	2	1.1
Lymphovascular invasion		
Yes	43	24.7
No	130	74.7
Unknown	1	0.5
Ki-67		
≤13%	59	34
>13%	108	62
Unknown	7	4
Estrogen receptors		
Positive	142	81.3
Negative	31	17.8
Unknown	1	0.5
HER2		
Positive	24	13.8
Negative	147	84.5
Unknown	3	1.7
Axillary node status^*^		
Positive	66	37.9
Negative	108	62.1
Breast cancer intrinsic subtype		
Luminal A	57	32.8
Luminal B HER2 –	68	39.1
Luminal B HER2 +	13	7.5
Pure HER2 +	10	5.7
Triple negative	19	10.9
Unknown	7	4

As we recently published, serum VEGF-C levels were normally distributed, with a median value of 6561.5 pg/mL (interquartile range = 2121.3 pg/mL). No significant correlation was found between serum VEGF-C levels and sentinel lymph node involvement (*p* = 0.626). There was no association between serum VEGF-C levels and the total number of affected lymph nodes (*p* = 0.146) or the number of involved axillary lymph nodes observed in the ALND surgical specimen obtained following a positive SLNB (*p* = 0.95). VEGF-C levels did not correlate with any of the other clinical or pathological variables [6]. However, there was a correlation between VEGF-C levels and the total number of platelets (*r* = 0.158, *p* = 0.037), as already reported in other studies [8].

**Figure 1:**
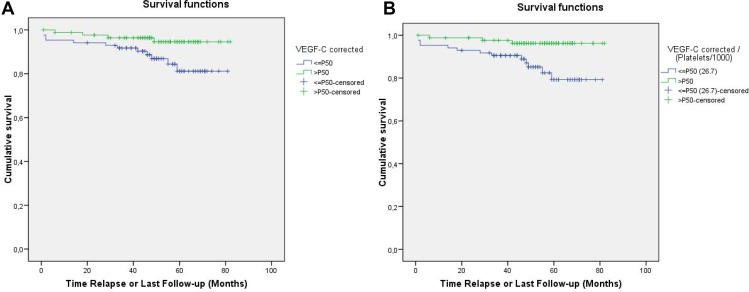
Relapse-free survival according to the median serum VEGF-C level (**A**) or the median VEGF-C level divided by the platelet count (**B**).

During a median follow-up period of 58 months (range 40–80), 16 relapses (9.2%) were observed. Three patients had local or regional relapses and 13 patients had distant metastases. There were also six deaths (3.4%), and three patients were censored because they were lost to follow-up. The five-year RFS and OS rates were 90.6% and 96.5%, respectively. Of the 16 relapses, only three occurred in patients with serum VEGF-C levels above the median. Preoperative serum VEGF-C levels were significantly associated with RFS. As shown in Figure 1A, the five-year RFS was higher in patients with VEGF-C levels above the median than in patients with lower VEGF-C levels (95.3% *vs.* 85.9%, *p* < 0.04). Likewise, Table 2 displays the RFS according to different percentiles of serum VEGF-C levels, again demonstrating that the RFS was better for patients with VEGF-C levels above the 75th percentile compared to patients with levels below the 75th percentile, although the results did not reach statistical significance (RFS 97.7%, *p* = 0.064).

**Table 2:** Five-year disease-free survival according to VEGF-C levels and VEGF-C levels divided by platelet count (VEGF-C/P)

	*P* ≤ 50	*P* > 50	*p*
VEGF-C	85.9%	95.3%	*0.034*
VEGF-C/P	84.7%	96.5%	*0.007*
	***P* ≤ 25**	***P* > 25**	
VEGF-C	88%	91.5%	0.464
VEGF-C/P	81%	93.8%	*0.008*
	***P* ≤ 75**	***P* > 75**	
VEGF-C	88.3%	97.7%	0.064
VEGF-C/P	89.1%	95.3%	0.205

**Figure 2:**
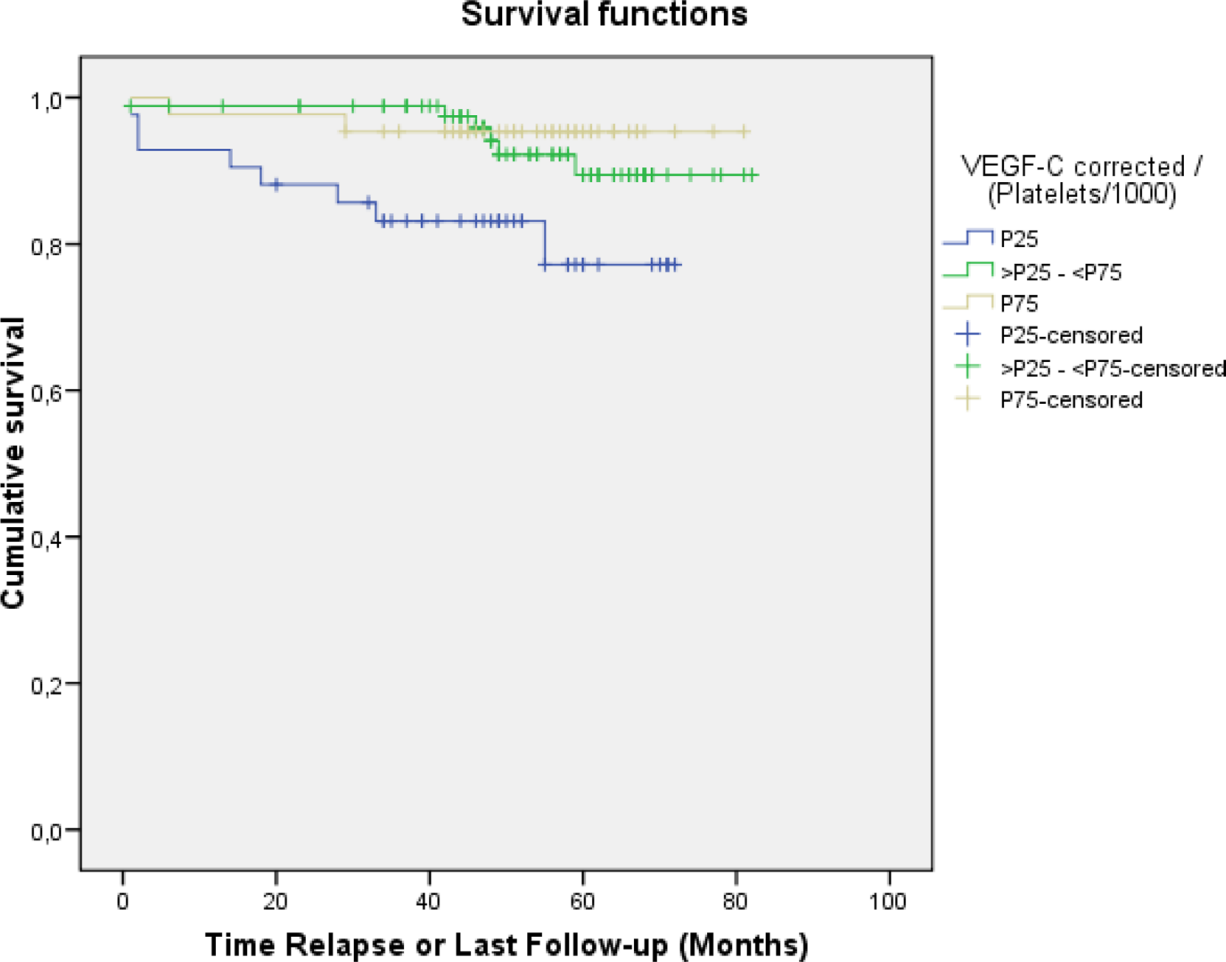
Relapse-free survival according to the percentile of the serum VEGF-C level divided by the platelet count

Given the correlation between the level of VEGF-C and the number of platelets [6], the VEGF-C/platelet count ratio and its association with RFS were analyzed. As shown in Figure 1B, patients with VEGF-C/platelet count ratios above the median had better five-year RFS than those whose ratios were lower (96.5% *vs.* 84.7%, *p* = 0.0007). Among the percentiles of patients shown in Table 2, those below the 25th percentile of VEGF-C/platelet count ratios had the lowest RFS (81%, *p* = 0.008) (Figure 2).

**Figure 3:**
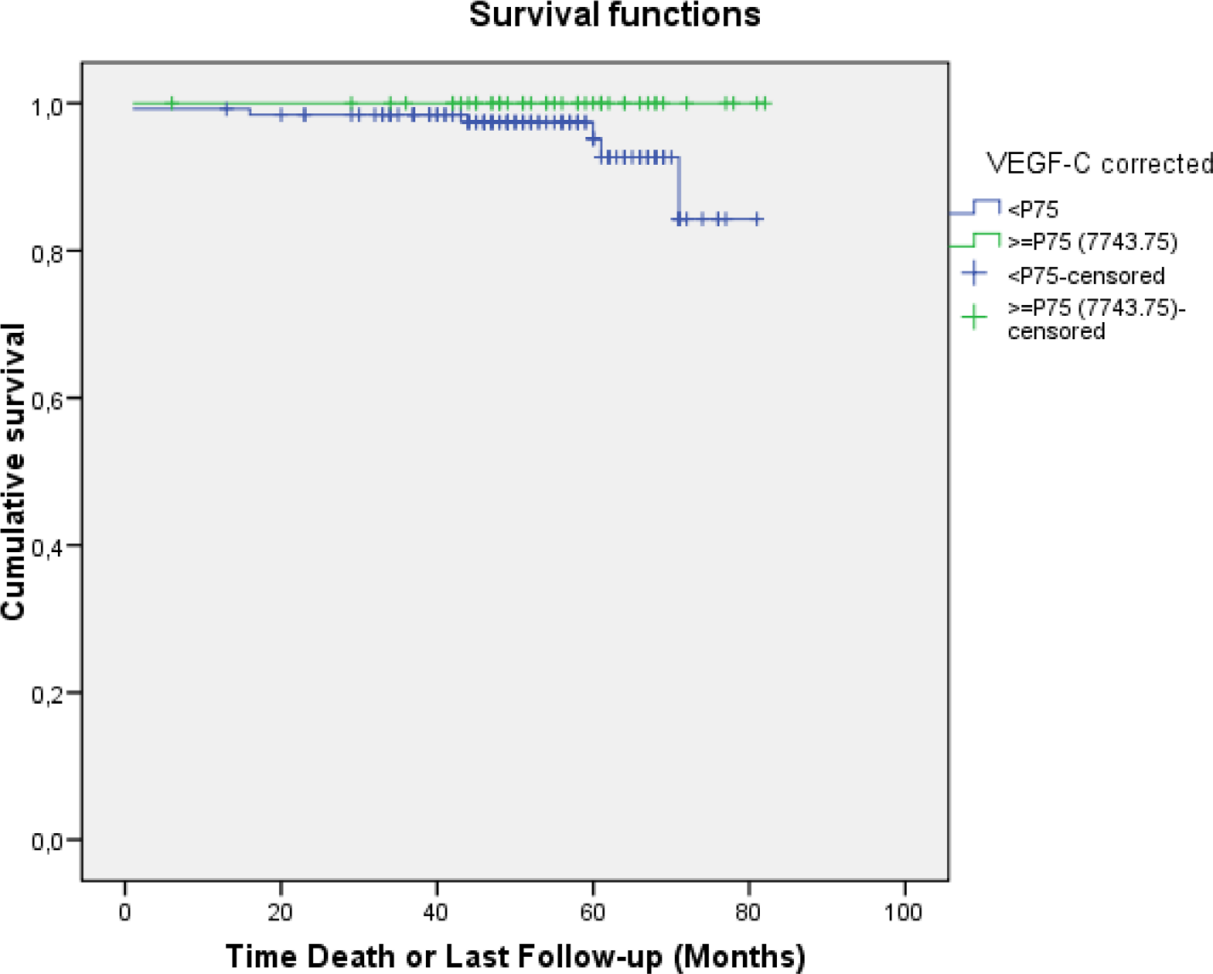
Overall survival according to the serum VEGF-C level (above or below the 75th percentile)

No association was found between VEGF-C levels and OS, possibly because of the low number of events. However, to date, none of the patients with serum VEGF-C levels above the 75th percentile (>7743.75 pg/mL) have died (five-year OS of 100% versus 95.3% for patients with VEGF-C levels ≤ P75; *p* = ns) (Figure 3).

Apart from serum VEGF-C levels, axillary lymph node involvement was the only prognostic factor associated with RFS. The five-year RFS was 95% in patients without axillary lymph node involvement, but was 86% in patients with pathological involvement of axillary lymph nodes from breast cancer (*p* = 0.044). As a result, we performed a multivariate analysis using a Cox regression model in which the following pathological variables were included: axillary lymph node involvement, tumor size, KI67 expression, estrogen receptor expression and serum VEGF-C levels (Table 3). Axillary lymph node involvement was the only negative independent prognostic factor for RFS in this model, with a HR of 2.9 (*p* = 0.05). Next, the multivariate analysis was performed again with the following prognostic factors: axillary lymph node involvement, tumor size, KI67 expression, estrogen receptor expression and VEGF-C levels corrected for the number of platelets. In this second analysis, the VEGF-C level corrected for the number of platelets (above the 75th percentile) was the only independent prognostic factor for RFS, with a HR of 3.9 (*p* < 0.03) (Table 3).

**Table 3:** Multivariate analysis

Model 1	RR	*p*	Model 2	RR	*p*
VEGF-C < median	2.5	0.07	***VEGF-C/P < median***	***3.9***	***0.03***
***Positive nodes***	***2.9***	***0.05***	Positive nodes	2.5	0.09
T > 2 cm	0.83	0.48	T > 2 cm	1.02	0.28
Ki 67 > 13%	1.3	0.29	Ki 67 > 13%	1.5	0.19
ER negative	1.5	0.16	ER negative	0.95	0.3

We also explored the association between serum VEGF-C levels and the different intrinsic breast cancer subtypes determined immunohistochemically following the St. Gallen 2011 criteria [9]. The distribution of subtypes was similar to that reported in the literature (Table 1). Serum VEGF-C levels did not differ significantly among patients with different intrinsic subtypes (*p* = 0.932). However, VEGF-C levels above the median remained a positive prognostic factor for RFS after adjustment for tumor phenotype, with a HR of 0.72 (95% CI 0.53–0.97, *p* < 0.04).

## DISCUSSION

Axillary lymph node metastasis is the main predictor of survival in patients with operable breast cancer. Many clinical and experimental results suggest that lymphangiogenesis facilitates tumor cell migration into lymphatic vessels [10, 11]. The main protein regulating lymphangiogenesis is VEGFR-3 [12], which is primarily expressed by lymphatic endothelial cells. The major ligand activating the VEGFR-3 pathway is VEGF-C, which is expressed in tumor cells and in macrophages and stromal cells of the tumor microenvironment [12, 13]. VEGF-C expression in breast cancer is associated with lymphatic vessel proliferation, lymphatic cell migration, tumor cell dissemination to the sentinel lymph node and other regional lymph nodes, tumor cell entry into the bloodstream through the thoracic duct, and lung metastasis formation [13].

Despite the growing experimental evidence of the involvement of VEGF-C in breast cancer progression, the prognostic value of VEGF-C overexpression remains controversial. VEGF-C expression in tumor tissue has been evaluated immunohistochemically in many studies, but the results have not been consistent [14]. However, a recent meta-analysis that included 21 studies and 2828 patients with breast cancer reported HRs of 1.87 (95% CI 1.25–2.79, *p* = 0.001) for RFS and 1.96 (95% CI 1.15–3.31, *p* = 0.001) for OS for patients with high tumor VEGF-C expression [5]. More recent studies have confirmed the negative prognostic role of VEGF-C based on its mRNA levels in tumor tissue [15, 16].

In the present prospective study, we analyzed the prognostic role of serum VEGF-C levels in patients with early breast cancer without clinical or radiological evidence of axillary lymph node metastasis. Overall, the enrolled patients had excellent prognoses based on conventional clinicopathological factors: over 82% of the patients had tumors smaller than 2 cm, two thirds did not exhibit tumor involvement of the sentinel lymph node and 81% expressed estrogen receptors (Table 1). Despite the fact that only 16 relapses were recorded during the follow-up period, patients with high serum VEGF-C levels were found to have a better prognosis than those with lower levels, to the extent that almost 98% of patients with VEGF-C levels above the 75th percentile did not relapse during the first five years of follow-up (no deaths were recorded in this group).

Only two studies have analyzed the prognostic value of circulating VEGF-C levels in breast cancer. In the first one, no correlation was found between plasma VEGF-C levels and OS in 122 patients [7]. However, in the other study, Gisterek *et al.* reported that serum VEGF-C levels above 1784.699 pg/mL predicted better four-year OS than lower levels in 349 patients (93% *vs.* 82%, *p* < 0.007) [8]. The latter study included operable patients with more advanced disease than the patients in our present study: one third of the patients had tumors greater than 2 cm, and over one third had lymphadenopathies with clinical signs of malignancy before surgery. The mean value of serum VEGF-C was 3040 pg/mL in patients with breast cancer, and did not differ from the value observed in women with benign breast disease. Unfortunately, the authors did not specify whether the study was designed prospectively, explain why they established the serum VEGF-C level cut-off point of 1784.699 pg/mL as significant for survival, or provide data regarding RFS.

Our group has already reported the positive prognostic implications of high serum VEGF-C levels for lymphoma patients. Among 88 patients with advanced-stage diffuse large B-cell lymphoma, those with high baseline serum VEGF-C levels had significantly greater four-year OS and progression-free survival (PFS) than those with lower levels. Major differences in survival were also found in patients with serum VEGF-C levels above the 75th percentile (>5755 pg/mL). The four-year PFS was 80% and the four-year OS was 95% in these patients, versus 53% and 68%, respectively, in patients with lower serum VEGF-C levels (*p* < 0.04 for RFS and *p* < 0.02 for OS) [17]. Similar results were obtained in 54 patients with Hodgkin lymphoma, as serum VEGF-C levels below the median (7675 pg/mL) were associated with worse PFS than levels above the median (66.7% *vs*. 88.5%; *p* = 0.064), although the difference did not reach statistical significance [18].

The biological mechanism explaining why the prognostic role of VEGF-C in operable breast cancer differs when it is measured in tumor tissue (by immunohistochemistry or mRNA analysis) and when it is measured in peripheral blood is unknown. Several hypotheses could explain this. During VEGF-C synthesis, a 58-kDa precursor protein is formed first, which is subsequently modified by proteases, generating the mature 21-kDa form. It is unclear whether not-fully-processed protein forms are secreted into the extracellular environment or can be detected by ELISA [19, 20]. However, the antibody used in our study enabled the detection of mature forms of VEGF-C, unlike the antibodies used in immunohistochemical techniques that can only detect unprocessed forms of VEGF-C, as suggested by Bando *et al*. [21]. These authors obtained similar results to ours in a study in which intratumoral VEGF-C levels were measured in the tumor lysate supernatant by ELISA [21]. Further studies are necessary to investigate the biological differences between processed and unprocessed or partially processed forms of VEGF-C.

The existence of less active forms of VEGFR-3 in tumors with better prognoses could also explain our observations. VEGFR-3 exists in two alternatively spliced isoforms, a long form (VEGFR-3L) and a short form (VEGFR-3S) [22]. These isoforms differ in the phosphorylation of three distal tyrosine residues, suggesting that they have different signaling capacities [23]. In a study conducted in breast cancer patients, no differences were observed in the expression of either VEGFR-3 isoform between breast tumor tissue and normal breast tissue. However, VEGFR-3L was almost undetectable in node-positive patients, while VEGFR-3S was the main form present [24]. VEGFR-3S is also the predominant isoform in prostate cancer patients with lymph node involvement [25]. This suggests that VEGFR-3S is the predominant splice variant in the most aggressive tumor cells. Therefore, it could be hypothesized that tumors expressing the less efficient variants of VEGFR-3 (VEGFR-3L) produce dysfunctional lymphatic vessels displaying a retrograde draining pattern [26], thus hindering lymphatic dissemination of the tumor and increasing interstitial pressure. This, in turn, could increase VEGF-C levels (by a mechanism of positive feedback), as has been shown in patients with lymphedema secondary to axillary surgery in breast cancer [27]. Thus, patients with higher serum VEGF-C levels could have tumors with a lower dissemination capacity and a better prognosis.

The effects of VEGF-C on cytotoxic T lymphocytes could also explain the better outcomes of patients with elevated serum levels of VEGF-C. A recent study demonstrated that *VEGF-C* gene expression strongly correlated with CCL21 expression and T cell inflammation in human metastatic melanoma, and serum VEGF-C concentrations were associated with both T cell activation and expansion [28]. The authors proposed that VEGF-C potentiates immunotherapy by attracting naïve T cells through the binding of CCL21 to the CCR7 receptor on cytotoxic T lymphocytes. The elevation of CCL21 mediated by the expression of VEGF-C was observed only in the microenvironment of the primary tumor and not in the regional lymph nodes. This study also revealed that elevated serum VEGF-C levels correlated with higher response rates and significantly better PFS in patients with metastatic malignant melanoma treated with a checkpoint blockade [28].

Our study indicated that patients with early breast cancer with high serum VEGF-C levels had a better prognosis than those with lower levels. Although the sample size was not calculated for this purpose (the study was designed to predict sentinel lymph node involvement) and there were only 16 relapses, the results appear to be robust (they were confirmed in a multivariate analysis) and consistent with other published breast cancer studies [7, 8, 21]. However, further research is required to elucidate the underlying biological mechanism of this phenomenon.

## MATERIALS AND METHODS

The main goal of this study was to determine whether serum VEGF-C levels were associated with the status of axillary lymph nodes determined by SLNB in patients with early breast cancer. We also examined the association of VEGF-C levels with various known prognostic variables in early breast cancer. Determining the relationship between serum VEGF-C levels and survival (RFS and OS) was a secondary objective.

### Study design and patients

This was a prospective cohort study that included 174 patients diagnosed with early breast carcinoma between September 2009 and November 2012. All patients were evaluated by a multidisciplinary committee and treated at the Hospital Costa del Sol (Marbella, Spain). Eligible patients were women aged over 18 years who were diagnosed with early-stage invasive breast carcinoma according to the seventh edition of the Cancer Staging Manual of the American Joint Committee on Cancer [29]. Tumor size was assessed by ultrasound and mammography, and needed to be less than 3 cm for the patient to be included in the study. If the ultrasound examination produced conflicting results, the larger diameter was taken. No multicentric tumors were included. An additional requirement for inclusion was a negative clinical and ultrasound axillary assessment. If there were any doubts regarding the axillary status, fine-needle aspiration cytology under radiologic guidance was conducted. Chest radiographic examinations and blood test results were negative in all cases. The exclusion criteria were a history of contralateral or metachronous breast cancer, the presence of any allergy contraindicating selective SLNB, and a history of any prior neoplasm other than non-melanoma skin cancer.

This research was conducted in compliance with the Declaration of Helsinki, and the study protocol was approved by the Ethics Committee of Costa del Sol Hospital in Marbella. The protocol procedures were only performed in patients who had given their prior informed consent for the study.

### Sample collection, surgical procedure and adjuvant therapy

Before the surgical treatment, two 10-mL samples of peripheral blood were taken from each patient and processed within 2 hours of collection. Serum and plasma were obtained from each sample.

All included patients underwent surgery of the primary tumor, along with SLNB and/or axillary lymph node dissection (ALND). In all cases, we used isotope tracing to localize the sentinel node. When this technique was not effective, the lymph node status was assessed after the ALND, and these patients remained in the study. Until September 2011, patients with involvement of any sentinel lymph node received a standard ALND. However, after the publication of the Z0011 study results [30], patients with only microscopic sentinel lymph node involvement were not subjected to ALND; this was the case for 15 patients with sentinel lymph node micrometastasis identified in the biopsy. As the main goal of the present study was to determine whether serum VEGF-C levels correlated with axillary lymph node status as determined by SLNB, this change did not provide any reason to interrupt the recruitment of patients.

The remaining clinical and pathological variables required were obtained after a histological study of the surgical specimens, including radiological and histological tumor size, histological type, histological grade, vascular and lymphatic invasion, hormone receptor status, amplification of human epidermal growth factor receptor 2 (HER2) expression, KI67 expression, sentinel lymph node status, number of lymph nodes involved and type of involvement (isolated cells, microscopic or macroscopic lymph node metastasis, extracapsular fat invasion and, when an ALND was performed, involvement of the remaining lymph nodes). The methodology used for the histological study of surgical specimens to determine sentinel lymph node status and other pathological variables has been previously published [6].

All patients received adjuvant treatment (radiotherapy, hormone therapy, chemotherapy and anti-HER2 therapy) in accordance with the institutional standard of care treatment at the time when the study was conducted.

### Determination of serum VEGF-C levels

VEGF-C levels were measured in serum obtained by centrifugation of two peripheral blood samples for 15 min at 1000 × *g*. The serum was then separated into several aliquots and frozen at −80° C.

The Quantikine (R&D System, Minneapolis, Minnesota, US) VEGF-C assay is a sandwich-type immunoassay in which a monoclonal antibody specific for VEGF-C has been coated onto the surface of a microplate so that the VEGF-C molecules in the serum bind to the immobilized antibody. Following repeated washing to remove unbound substances, an enzyme-labeled polyclonal antibody specific for VEGF-C was added to the wells. After further washing, the samples were incubated with a substrate solution, and finally a stop solution was added, ending the reaction and producing a colored end product which was measured to determine the concentration of VEGF-C. The amount of bound conjugate was measured in units of absorbance at 540 or 570 nm. The absorbance was measured with a ZENIT SP+ analyzer (Menarini), which is an open system for processing immunoenzymatic tests using the ELISA 8Å~12 microplate system.

The results were calculated as the average of the duplicate readings for each standard, control and sample, and then the average optical density of the zero standard was subtracted. The optical density was mapped against the concentration of the standards and the best fit curve was drawn. The concentration of VEGF-C in each sample was determined by interpolation of the optical density data in the standard curve. If the sample was diluted, the concentration read from the standard curve was multiplied by the dilution factor. This procedure meets the requirements of Directive 98/79/EC on *in vitro* diagnostic medical devices.

Several studies have associated serum VEGF levels with platelet counts, as platelets appear to store these factors and release them into the bloodstream [8]. We therefore took into account the platelet counts obtained in the plasma samples.

### Statistical analysis

All data collected in this project were anonymized, in accordance with the Biomedical Research Act currently in force in Spain (Act 14/2007 of July 3), the national regulations on data protection (Act 41/2002 of November 14, Act 15/1999 of December 15) and the Declaration of Helsinki. The sample size required for the diagnostic test (serum VEGF-C) was predetermined for an expected prevalence of axillary lymph node positivity of 25%, with an absolute accuracy of 3%, a confidence level of 95% and an expected specificity of 95%. We calculated that a sample of 199 patients with operable breast cancer would be needed; thus, a minimum sample size of 200 patients was set.

A descriptive analysis was carried out with measures of central tendency, position and dispersion for quantitative variables, and of frequency distribution for qualitative variables. The Kolmogorov-Smirnov test was used to determine the normality of the samples for the quantitative variable VEGF-C. Univariate analyses were conducted with RFS as the main outcome variable and serum VEGF-C levels as the main independent variable. The relationship between two quantitative variables was assessed with Pearson's correlation. The relationship between a quantitative variable and a qualitative variable was assessed with the Mann–Whitney *U* test for qualitative dichotomous variables, or with non-parametric one-factor analysis of variance for qualitative variables with three or more categories. The association between two qualitative variables was assessed by the chi-square test.

The probability of relapse or death was estimated with the Kaplan-Meier method, and differences between the patient groups were assessed with the log-rank test. Finally, multiple logistic Cox regression was performed with the occurrence of relapse or death as the outcome variable. The variables found to be significant in the bivariate analysis were included in the model by the stepwise forward method, and the HRs were determined with the corresponding 95% CIs. The significance level was set at *p* < 0.05 for all the analyses.
